# Medication Refill Adherence and Mortality Risk in the Oldest-Old:

**DOI:** 10.1093/geroni/igaf122.668

**Published:** 2025-12-31

**Authors:** Raymond Kheirbek, Brock Beamer, Patrick McArdle, Nadim Ojaimi, Bernard Davies-Teye, Abree Johnson, Farrokh Alemi, Raya Kheirbek

**Affiliations:** Johns Hopkins University, Baltimore, Maryland, United States; University of Maryland, Baltimore, Maryland, United States; University of Maryland, Baltimore, Maryland, United States; University of Maryland, Baltimore, Maryland, United States; University of Maryland School of Pharmacy, Baltimore, Maryland, United States; University of Maryland School of Pharmacy, Baltimore, Maryland, United States; George Mason University, Fairfax, Virginia, United States; University of Maryland School of Medicine, Baltimore, Maryland, United States

## Abstract

**Background:**

The U.S. population aged 85 and older is growing rapidly, yet little research explores how medication refill adherence affects mortality in this group. Adherence is critical for managing hypertension and reducing cardiovascular risk, but missed refills may signal broader health, cognitive, or social issues beyond non-compliance.

**Objective:**

This study examines the link between medication refill adherence and all-cause mortality in Medicare beneficiaries aged 85+ with hypertension. Design, Setting, and Participants: This retrospective cohort study analyzed Medicare claims data (2006–2021), using a 5% sample (2006–2016) and a 20% sample (2017–2021) of beneficiaries aged 85+ with confirmed hypertension.

**Exposure:**

Refill patterns for antihypertensive drugs, statins, and proton pump inhibitors (PPIs) were evaluated via prescription claims, categorizing participants into regular vs. missed refill periods.

**Outcome Measure:**

All-cause mortality was the primary outcome, with mortality risk compared between refill patterns using Cox proportional hazards models with time-varying exposure.

**Results:**

Regular antihypertensive refills were linked to a 55-66% lower mortality risk (HR: 0.336–0.451), consistent even in those aged 95 + (HR: 0.366–0.441). Similar mortality reductions were seen with statins and PPIs, suggesting refill adherence reflects overall health stability rather than antihypertensive effects alone.

**Conclusion:**

Missed refills strongly predict higher mortality in the oldest-old, likely indicating cognitive decline, frailty, or social vulnerability rather than purely pharmacologic factors. Pharmacy refill data could serve as an early-warning tool to identify at-risk older adults for targeted interventions. Further studies should investigate causal pathways and policy implications.

